# Burnout among rural hospital doctors in the Western Cape: Comparison with previous South African studies

**DOI:** 10.4102/phcfm.v10i1.1568

**Published:** 2018-05-24

**Authors:** Andrew R. Liebenberg, Johan F. Coetzee, Hofmeyr H. Conradie, Johan F. Coetzee

**Affiliations:** 1 Division of Family Medicine and Primary Care, Stellenbosch University, South Africa; 2 Ukwanda Centre for Rural Health, Stellenbosch University, South Africa; 3 Department of Anaesthesia and Critical Care, Stellenbosch University, South Africa

## Abstract

**Background:**

Burnout among doctors negatively affects health systems and, ultimately, patient care.

**Aim:**

To determine the prevalence of burnout among doctors working in the district health system in the Overberg and Cape Winelands districts of the Western Cape Province and to compare the findings with those of previous South African studies.

**Setting:**

Rural district hospitals.

**Methods:**

During 2013, a validated questionnaire (Maslach Burnout Inventory) was sent to 42 doctors working in the district health system within the referral area of the Worcester Hospital, consisting of the Overberg health district and the eastern half of the Cape Winelands.

**Results:**

Response rate was 85.7%. Clinically significant burnout was found among 81% of respondents. High levels of burnout on all three subscales were present in 31% of participants. Burnout rates were similar to those of a previous study conducted among doctors working in the Cape Town Metropolitan Municipality primary health care facilities. Scores for emotional exhaustion (EE) and depersonalisation (DP) were greater than those of a national survey; however, the score for personal accomplishment (PA) was greater. EE and PA scores were similar to that of a study of junior doctors working in the Red Cross Children’s Hospital; however, EE was smaller.

**Conclusion:**

This study demonstrates high burnout rates among doctors working at district level hospitals, similar to the prevalence thereof in the Cape Town Metropolitan primary health care facilities. Health services planning should include strategies to address and prevent burnout of which adequate staffing and improved work environment are of prime importance.

## Introduction

An increasing body of evidence suggests that physician burnout has a detrimental effect on health care delivery worldwide.^1^ Burnout among doctors negatively influences recruitment and retention of doctors, effectiveness and efficiency of health systems and, ultimately, patient care.^2^ These are critical issues for the District Health System of South Africa. Maslach’s burnout model defines a syndrome of emotional exhaustion (EE), depersonalisation (DP) and/or a low sense of personal accomplishment (PA) that is related to prolonged occupational stress and frustration and results in reduced effectiveness at work.^2,3^ Emotional exhaustion relates to the occupational stress dimension of burnout, while DP is a consequence of EE and refers to job detachment and callousness, which is detrimental to the doctor–patient relationship. Personal accomplishment refers to perceived effectiveness and efficiency at work. Physician burnout results in absenteeism, increased job turnover, cynicism and low job satisfaction^2^. There is also a strong association between burnout and substance abuse, relationship problems and depression.^3^

There are both contextual and individual factors related to burnout, of which contextual (situational) factors appear to play the predominant role.^2^ Contextual factors include organisational or management structure and style, workload, sleep deprivation, resources, financial compensation, practice setting, patient characteristics, career progression opportunities, work team and community, communication and feedback.^2, 3^ Individual factors include demographics, personality type, external locus of control, job dissatisfaction, and level of social support.^2,3^

Globally, burnout rates among doctors range between 25% and 60%.^2^ In 1994, Schweitzer found a 77.8% burnout rate among young doctors working within the South African district health service.^4^ Subsequent South African studies found similarly high levels of burnout.^5,6,7^ With regard to the Western Cape Province, Rossouw et al. reported that 76% of doctors working within the provincial primary health care system of the Cape Town Metropole (CTM) experienced clinically significant levels of burnout.^6^ Doctors of all ages and demographic profiles are prone to burnout, but junior doctors (including registrars) are particularly vulnerable and burnout levels are particularly high among doctors working in the primary health care environment.^2,3,4,5,8^ No published information is available concerning burnout among district hospital doctors practising in rural areas in the Western Cape. The primary purposes of this cross-sectional study were to determine the prevalence of burnout among doctors working within the Overberg and Cape Winelands district health systems of the Western Cape Province and to compare the findings with those of a normative study^9^ as well as three previous South African studies.^5,6,7^ One study, that is, a 2003 national survey by Peltzer and colleagues of 402 doctors randomly selected from the Health Profession Council’s register of 27 551 medical practitioners working in both the private and state sectors, was regarded as a South African normative study. The secondary aim was to identify from the available literature methods for preventing and treating burnout by means of individual-focused approaches and organisational strategies, with special reference to the Western Cape Province of South Africa.

## Research methods and design

### Study design

An anonymised survey (prospective cross-sectional study).

### Setting

District hospitals of the Overberg District (Caledon, Hermanus, Bredasdorp and Swellendam hospitals) and the Cape Winelands District (Ceres, Robertson and Montagu hospitals).

### Study population and sampling strategy

The study population consisted of doctors working at the district hospitals of the Overberg and Cape Winelands (OB & CW) districts. At the time of the study, these hospitals ranged in size from 30 to 80 beds. All seven hospitals have a 24 h emergency centre, labour ward and theatre service. The hospitals also supply doctor outreach support to the referring clinics. Each hospital was staffed by between 3 and 10 doctors. The study was limited to doctors working in district hospitals and excluded doctors based in community health centres and clinics. Only doctors participating in commuted overtime were included.

### Data collection

The Maslach Burnout Inventory–Human Services Survey (MBI-HSS)^9^ consists of 22 statements concerning personal feelings and attitudes that are responded to on a Likert scale that ranges from 0 = never to 6 = every day. The inventory is designed to identify the frequency and intensity of symptoms that comprise the three dimensions of the burnout syndrome (EE, DP and PA). The graded MBI-HSS scoring scale represents the upper, middle and lower thirds of scores of a normative sample for medical human service occupations.^9^ Clinically significant burnout is defined as a high score for either EE or DP subscales (Table 1).^9^

**TABLE 1:** Classification of Maslach Burnout Inventory–Human Services Survey Burnout component scores.

Burnout level	Emotional exhaustion	Depersonalisation	Personal accomplishment
High	≥ 27	≥ 10	0–33
Moderate	19–26	6–9	34–39
Low	0–18	0–5	≥ 40

*Source*: Maslach D, Jackson S, Leiter M, Schaufeli W, Schwab R. Maslach Burnout Inventory manual, general survey, human services survey, educators survey and scoring guides. Menlo Park, CA: Mind Garden; 1986.

The MBI-HSS questionnaires were distributed to 42 community service medical officers (CSMOs), medical officers (MOs), family medicine registrars and specialist family physicians (FPs) who met the inclusion criteria between 20 February and 31 March 2013. The MBI-HSS is self-explanatory and takes less than 30 min to complete. To protect anonymity within small hospital teams, age, gender and years of experience were not recorded. The completed questionnaires were scored and categorised using the MBI-HSS Scoring Key, which scores participants on each of the three subscales (EE, DP and PA).^9^ EE and DP scores correlate directly with burnout levels, while the PA subscale is inversely proportional to the level of burnout.

### Data analysis

Statistical analysis was performed using Medcalc software^10^ and Confidence Interval Analysis software.^11^ Scores were regarded as numerical (interval) data. The Shapiro–Wilk test and coefficients of skewness and kurtosis were used to determine whether the data were normally distributed. Intergroup comparisons between specialist FPs and MOs were done using two-sided *t*-tests for independent samples, provided the criteria for normal distribution and equal variances (Levene test) were satisfied. *T*-Tests were used to compare scores with those of the normative study of Maslach et al.^9^ and with two previous South African studies^5,7^ using the published means and standard deviations, on condition that the F-test for equal variances was not significant. The Bonferroni correction for multiple comparisons was applied. Confidence intervals for proportions and for differences between proportions were calculated using the Wilson and Newcombe methods, respectively.^12^ The scales of the Maslach Burnout Inventory scores are not widely understood clinical measurements such as blood pressure, blood glucose, etc. therefore, 95% confidence intervals of the differences between mean values are difficult to interpret. In order to elucidate the effect size of the differences between the studies, we calculated Cohen’s *d*, an unit-independent measure of effect size,^13^ as follows:

$$d=\frac{\left|m_{A}-m_{B}\right|}{\sigma }$$ [Eqn 1]

where *m*_*A*_ and *m*_*B*_ are the two means and σ is the pooled standard deviation. Thus, Cohen’s *d* indicates by how many standard deviations two means differ.

Participants’ scores were categorised into three degrees of burnout, high, moderate and low, according to the grouping displayed in Table 1.^9^ Comparisons of proportions between this study and that of the CTM study^6^ were done using the Fisher’s exact test. The small numbers comprising this study did not permit meaningful comparisons between individual hospitals. An alpha value of ≤ 0.05 was accepted as indicating statistical significance.

### Ethical considerations

Ethics approval was obtained from the University of Stellenbosch Health Research Ethics Committee (reference No. N11/09/278). Demographic details of the participants were not recorded in order to ensure their anonymity.

## Results

### Burnout scores

**FIGURE 1:**
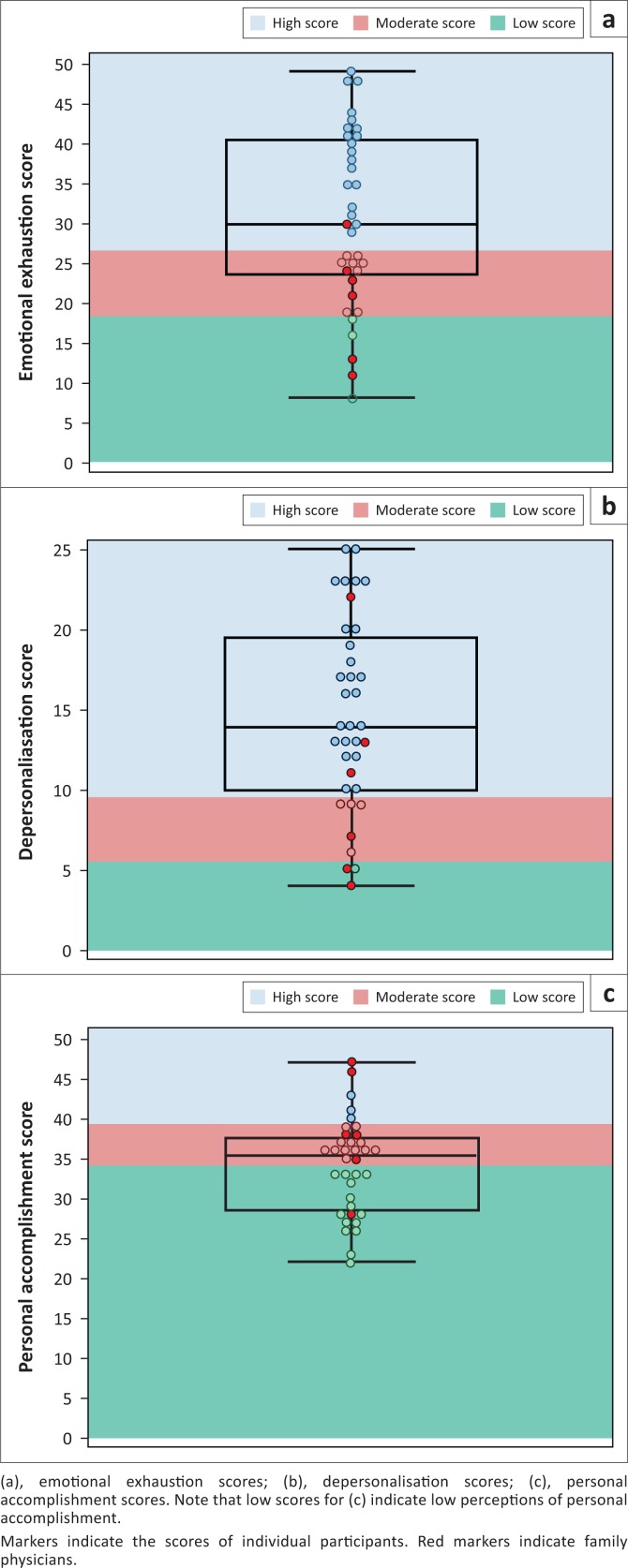
Box and whisker plots of the three constituents of burnout among rural district hospital doctors.

**FIGURE 2:**
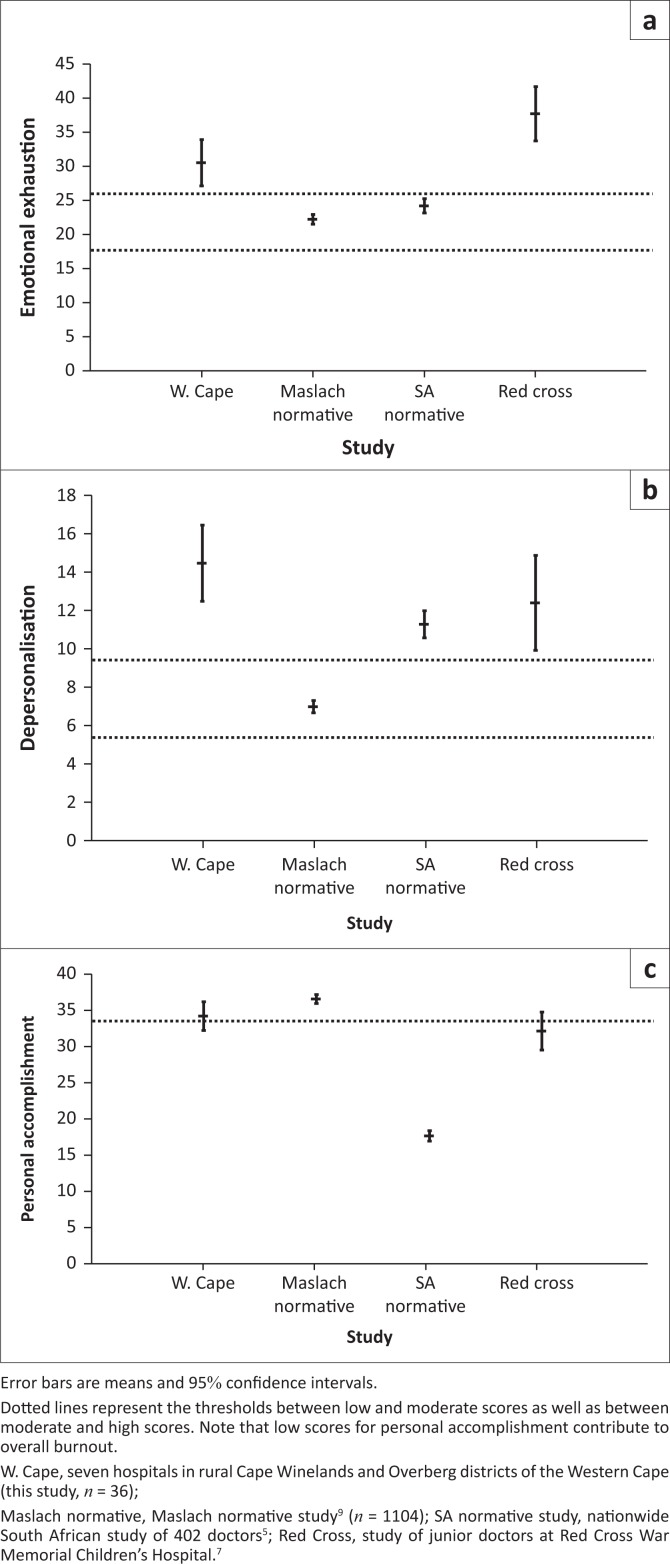
Error bar diagrams comparing burnout scores.

Thirty-six of 42 eligible doctors from seven hospitals participated, including all six FPs (with a response rate of 85.7%). The scores for the 36 participants are presented in Table 2^5,7,9^ and Figure 1. High mean scores for either EE or DP indicated the presence of clinically important levels of burnout. Mean scores for EE and DP and the lower bounds of their 95% confidence intervals were within the high score categories. These scores were significantly greater than the normative scores and had large Cohen’s *d* effect sizes. Mean PA scores did not differ from normative values with low effect size. Table 2^5,7,9^ and Figure 2 present comparisons with two previous studies of South African doctors which employed the MBI-HSS questionnaire. Mean EE, DP and PA scores were significantly greater than those of a national (normative) survey.^5^ Comparing a study of junior doctors working in the Red Cross Children’s Hospital,^7^ it was found that while rural doctors’ EE scores were significantly less, DP and PA scores did not achieve statistical significance and had low values for Cohen’s *d*. Rural MOs had a significantly greater mean EE than rural FPs and a lower mean PA, but mean DP scores did not differ (Table 3).

**TABLE 2:** Maslach Burnout Inventory–Human Services Survey scores for the three components of burnout: Comparison with the normative and two previous South African studies.

Variables	OB & CW *N* = 36	Maslach normative study *N* = 1104		National study *N* = 402		Red Cross Children’s Hospital *N* = 22	
	Mean (s.d.) 95% CI	Mean (s.d.) 95% CI	*p* [Cohen’s *d*]	Mean (s.d.) 95% CI	*p* [Cohen’s *d*]	Mean (s.d.) 95% CI	*p* [Cohen’s *d*]
Emotional exhaustion	30.5 (11.0) 27 to 34	22.2 (9.5) 21.6 to 22.8	< 0.0001 [0.9]	24.2 (10.8) 23 to 25	0.0009 [0.6]	37.7 (8.9) 33.8 to 41.7	0.012 [0.7]
Depersonalisation	14.6 (6.0) 13 to 17	7.1 (5.2) 6.8 to 7.4	< 0.0001 [1.4]	11.4 (6.7) 10.7 to 12.1	0.0060 [0.5]	12.6 (5.6) 10.1 to 15.1	0.212 [0.3]
Personal accomplishment	34.1 (6.0) 32 to 36	36.5 (7.7) 36 to 37	0.0640 [0.3]	17.4 (6.8) 16.7 to 18.1	< 0.0001 [2.5]	32.1 (5.8) 29.5 to 34.7	0.218 [0.3]

*Source*: Peltzer K, Mashego TA, Mabeba M. Short communication: Occupational stress and burnout among South African medical practitioners. Stress Health. 2003;19(5):275–280. https://doi.org/10.1002/smi.982; Stodel JM, Stewart-Smith A. The influence of burnout on skills retention of junior doctors at Red Cross War Memorial Children’s Hospital: A case study. S Afr Med J. 2011;101(2):115–118. https://doi.org/10.7196/SAMJ.4431; Maslach D, Jackson S, Leiter M, Schaufeli W, Schwab R. Maslach Burnout Inventory manual, general survey, human services survey, educators survey and scoring guides. Menlo Park, CA: Mind Garden; 1986.

Note applying the Bonferroni correction for multiple comparisons, alpha = 0.0167 to accept statistical significance.

High burnout scores: emotional exhaustion > 27; depersonalisation > 10; personal accomplishment < 33.

Interpretation of Cohen’s *d* estimation of effect size: very small 0.01; small 0.20; medium 0.50; large 0.80; very large 1.20; huge 2.0.

OB & CW, study of seven hospitals in the rural Overberg District and Cape Winelands District of the Western Cape (this study); *N*, number of subjects studied; s.d., standard deviation; 95% CI, 95% confidence interval; *p, p*-value for the *t*-test for independent samples, comparison with OB & CW study; 95% CI Diff, 95% confidence interval for the difference between mean values.

**TABLE 3:** Burnout scores: Comparison between medical officers and specialist family physicians.

Variables	Medical officers *N* = 30			Family physicians *N* = 6			*p*	95% CI Diff
	Mean	s.d.	95% CI	Mean	s.d.	95% CI [*d*]		
Emotional exhaustion	33	11	29 to 36	20	7	13 to 28 [1.2]	0.011	3 to 21
Depersonalisation	16	6	13 to 18	10	7	3 to 17 [0.9]	0.053	−0.08 to 10.4
Personal accomplishment	33	5	31 to 35	39	7	31 to 46 [1.0]	0.037	0.3 to 10.7

High burnout scores: emotional exhaustion > 27; depersonalisation > 10; personal accomplishment < 33.

Interpretation of Cohen’s *d* estimation of effect size: very small 0.01; small 0.20; medium 0.50; large 0.80; very large 1.20; huge 2.0.

s.d., standard deviation; 95% CI, 95% confidence interval; 95% CI Diff, 95% confidence interval of the difference between the mean values; *p, p*-value for *t*-test for independent samples; *d*, Cohen’s *d*.

### Analysis of proportional data

**FIGURE 3:**
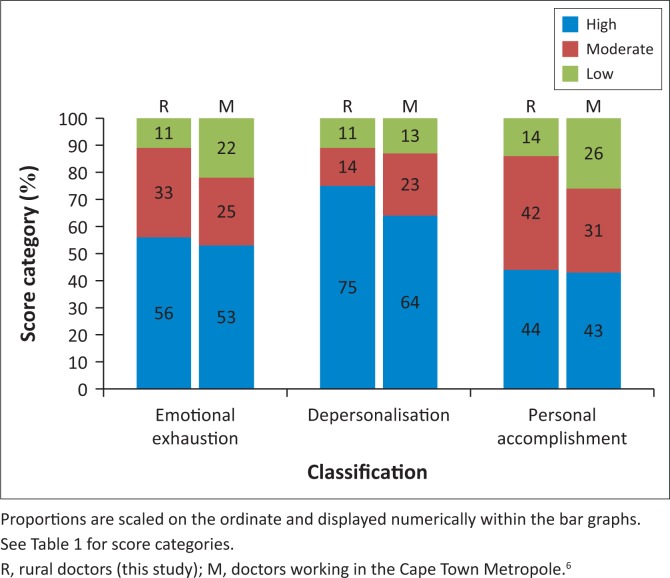
Proportions of doctors who scored in the high, moderate and low categories for the three constituents of the Maslach Burnout Inventory.

Figure 3 and Table 4^6^ portray the proportions of participants in our study and in the CTM study who had high, moderate and low degrees of burnout according to the three subscales. In our study, 81% (95% CI 65% to 90%) of participants demonstrated high EE or DP scores, either of which is a measure of clinically significant burnout. High scores for both EE and DP were present in 50% (95% CI 35% to 66%) of participants. No doctor had low burnout levels in all three subscales. Three FPs and one MO did not have a high burnout level on any subscale. An analysis of the combinations of various subscales comprising the three burnout subscales is presented in Table 4.^6^ None of the proportions differed significantly from those of the CTM study (*p* = 0.34–0.66).

**TABLE 4:** Analysis of the prevalence of various burnout subscales for the three components of burnout: Comparison with the findings of Rossouw et al. for the Cape Town Metropole.

Burnout score range	OB & CW (*N* = 36)	Cape Town Metropole (*N* = 132)	*p*	95% CI Diff
High for any one category (EE, DP or PA)	32 (89%) 75% to 96%	111 (84%) 76% to 89%	0.602	−10% to 15%
High for EE or DP	29 (81%) 65% to 90%	100 (76%) 68% to 82%	0.659	−12% to 17%
High for EE and DP	18 (50%) 35% to 66%	55 (42%) 34% to 50%	0.449	−9% to 27%
High for EE, DP and PA	11 (31%) 18% to 47%	-	-	-
No high level for any category	4 (11%) 4% to 25%	21 (16%) 11% to 23%	0.602	−10% to 15%
Low level for all three categories (EE, DP and PA)	0 (0%) 0% to 1%	6 (5%) 2% to 10%	0.343	−5% to 10%

*Source*: Rossouw L, Seedat S, Emsley RA, Suliman S, Hagemeister D. The prevalence of burnout and depression in medical doctors working in the Cape Town Metropolitan Municipality community healthcare clinics and district hospitals of the Provincial Government of the Western Cape: A cross-sectional study. S Afr Fam Pract. 2013;55(6):567–573. https://doi.org/10.1080/20786204.2013.10874418

Data are presented as number (percentage), 95% confidence interval of the percentage.

OB & CW, study of seven hospitals in the rural Overberg District and Cape Winelands District of the Western Cape (this study); EE, emotional exhaustion; DP, depersonalisation; PA, personal accomplishment.

*p, p*-value for Fisher’s exact test; 95% CI Diff, 95% confidence interval for the difference between proportions (rural vs. Cape Town Metropole).

## Discussion

This study revealed a high prevalence of burnout among OB & CW hospital doctors including specialist FPs, MOs and CSMOs. Comparisons with the Maslach^9^ and Peltzer^5^ normative studies revealed that the differences in mean EE and DP scores achieved not only statistical significance, but they are also of meaningful clinical importance as indicated by their widely separated confidence intervals and large effect sizes (Cohen’s *d*). Interestingly, mean PA did not differ from the Maslach normative mean score and was markedly greater than that of the Peltzer study^5^ which showed a remarkably low score for PA.

Our findings are similar to the recent study by Rossouw and co-workers^6^ among 132 district level doctors in the CTM who demonstrated equally high burnout rates with regard to prevalence and severity. Clearly physician burnout is a problem among urban and rural doctors working within the public sector primary health care system of the Western Cape.

Participants in the Peltzer study^5^ also completed Spielberger’s Job Stress Survey.^14^ Job stress predicted EE and DP. The greatest sources of job stress included working overtime, lack of organisational support, inadequate salary, making critical on-the-spot decisions and dealing with crisis situations, factors that have been identified in other publications.^1^ Rossouw and co-workers^6^ have listed the 10 most important factors that contribute to burnout in primary health care doctors (Table 5).^6^

**TABLE 5:** Factors that contribute to burnout in primary health care doctors.

Mean ranking†	Variable
1	Hours worked
2	Workload
3	Working conditions
4	Public system-related frustration
5	Work stress and anxiety
6	Balancing work and personal life
7	Vacation limit
8	Inadequate equipment
9	Lack of management support
10	Low work satisfaction

*Source*: Rossouw L, Seedat S, Emsley RA, Suliman S, Hagemeister D. The prevalence of burnout and depression in medical doctors working in the Cape Town Metropolitan Municipality community healthcare clinics and district hospitals of the Provincial Government of the Western Cape: A cross-sectional study. S Afr Fam Pract. 2013;55(6):567–573. https://doi.org/10.1080/20786204.2013.10874418

† , mean ranking of factors in order of perceived importance.

Family physicians appear to have fared better than MO’s, considering that the mean scores for EE and PA indicated statistically significantly lower burnout levels than those of the MOs (mean EE 20 vs. 33; PA 33 vs. 39 and Cohen’s *d* values indicative of large effect sizes). However, the 95% confidence intervals of the differences between the mean values were wide and included trivial differences as well as possibly clinically important differences. Furthermore, the mean DP scores did not achieve statistical significance; therefore, the clinical importance of these findings is uncertain. Certain individual FPs scored high on the EE and DP scales and low on the PA scale (Figure 1). Additional studies of larger sample sizes are required to elucidate any real differences as well as the extent of burnout that may be emerging among FPs. The Western Cape Healthcare 2030 document^15^ states that district hospitals will provide a FP-driven service; however, the appearance of signs of burnout among this small sample of family practitioners, whose role is *inter alia* mentoring of junior doctors, deserves investigation.

The Western Cape Department of Health has outlined its vision for the future in a document entitled ‘Healthcare 2030: The road to wellness’,^15^ an ambitious, wide-ranging plan to improve the quality of health care that is based upon ‘patient-centred quality of care’. With regard to human resources, the document recognises that:

staff satisfaction is directly related to improved patient satisfaction. Thus part of the 2030 strategy is geared to ensure that employees are engaged, empowered and happy to be at work that will in turn generate better outcomes for patients….^15^

In a section entitled ‘Improved Staff Experience’, the document acknowledges the ‘high levels of burnout among staff as a result of heavy workloads and a stressful working environment’ and that:

they experience dissatisfaction with the people-management skills of their line managers and do not believe that they are valued, feel listened to or cared for by the organisation and thus have limited engagement with the organisation.^15^

A goal is repeatedly stated that the Department of Health should become an employer of choice. Various approaches are offered that include improving management systems, a staff recognition system and an employee wellness programme. These intentions are encouraging. Nevertheless, it is noteworthy that whereas long working hours, high workload, working overtime and lack of organisational support feature prominently as causes of burnout in South African and overseas studies, the provision of adequate staff numbers appropriate to the clinical load is not specifically addressed in the document. The findings of previous reports over several decades regarding the plight of South African rural doctors were confirmed in 2004 by De Villiers and De Villiers^16^ who interviewed doctors from 20 Western Cape rural hospitals. The authors stated ‘Our findings confirmed that substantial after-hour duties, an excessive workload and a perceived lack of management support impact negatively on rural district hospital doctors’. It is possible that after 12 years the situation may not have improved.

Wallace and colleagues conducted an extensive review of high-quality studies regarding physician wellness^1^ and concluded that burnout and psychological distress are highly prevalent among primary care physicians. At the organisational level, excessive burnout, job stress, fatigue and dissatisfaction result in suboptimal patient care, increased clinical errors, reduced productivity and increased staff turnover. At personal level, burnt-out physicians are exposed to increased risks of needle-stick injuries and motor vehicle accidents as well as making serious medical errors.^17^ It is with these considerations in mind that the results of these studies need to be considered by district health policymakers. In view of the relationship between physician wellness and patient care outcomes, burnout levels should ideally be no greater than normative values. Considering local service requirements and the nature of the clinical work, it is inevitable that doctors working in district hospitals will at some time experience exhaustion or burnout. However, such high a prevalence of excessive levels of burnout that have appeared among primary health care doctors in the Western Cape is unacceptable and poses a threat to patient care as well as to the physical and mental health of the doctors.

Several studies have investigated the attitudes, behavioural traits and strategies employed by physicians who function successfully in stressful job situations known to result in burnout. Keeton and colleagues investigated factors determining career satisfaction, work–life balance and burnout among 935 physicians practising in specialities that predispose to burnout (general obstetrician–gynaecologists, subspecialty obstetrician–gynaecologists, general internal medicine, general paediatrics, general surgery and family medicine).^17^ They concluded that the capacity to exercise some control over work schedule and hours worked is the most important predictor of low burnout and satisfactory work–life balance. Low levels of burnout strongly predicted career satisfaction. A study of 8050 direct patient care physicians demonstrated that professional autonomy was more important than income in determining physician career satisfaction.^18^

Various strategies have been proposed to assist health care systems and health care workers develop coping mechanisms and resilience to the stresses that lead to burnout (Table 6).^16,17,18,19,20,21,22,23,24,25,26^ These include mindfulness interventions, mentoring programmes, etc., as well as improvement of the work environment by improving continuity of care, professional autonomy, etc. Recently, West and co-workers conducted a meta-analysis regarding the effectiveness of various interventions to reduce burnout among doctors.^27^ They included 47 studies consisting of 15 randomised clinical trials (716 physicians) and 37 cohort studies (2914 physicians). Effective organisational approaches included modified duty hour requirements and modifications to clinical work processes. Effective individual-focused strategies included mindfulness-based approaches, stress management training and small group curricula. Structural or organisational interventions were more effective than individual-focused ones. Overall, the incidence of high scores for EE was reduced by 14% (95% CI 11% to 18%) and that of high scores for DP was reduced by 4% (95% CI 0% to 8%).

**TABLE 6:** Strategies to assist health care workers in avoiding burnout.

Focus of strategy	Strategies mentioned
Increased resilience among individuals	Mentoring programmes^19^
	Developing life skills^19,20,21^ (Boundary setting, finding work–life balance and self-care)
	Reflective practice^21^
	Balint groups^22^
	Mindfulness training^19,23,24,25^
	Maintaining certain attitudes^19^ (acceptance and realism, self-awareness and reflexivity, recognising when change is necessary and appreciating the good things)
Improving the work environment and job satisfaction	Practising patient centredness^20^
	Increased continuity of care^18,20^
	Develop and support doctors to practise in their field of interest^16,19,20,26^
	Reduction of the administrative load of clinicians^16,19,20^
	Reduction of frustration of clinicians (ensuring that sufficient equipment and support is available)^19,20^
	Measuring burnout levels among clinicians and developing a strategy^19,20^
	Use of locums or contract posts during periods of annual leave or maternity leave of permanent staff^16^
	Appointing adequate staff numbers^16^
Increased professional autonomy	Allowing doctors to adjust working schedules and working hours^1,17,19,20,26^
	Allowing clinicians to plan their leave^17,19^ (allowing leave for birthdays, family responsibilities and participation in other interests)
	Use of flexible working hours according to peak patient load^17,19,26^

Note: Please see the full reference list of the article, Liebenberg AR, Coetzee JF Jr, Conradie HH, Coetzee JF. Burnout among rural hospital doctors in the Western Cape: Comparison with previous South African studies. Afr J Prm Health Care Fam Med. 2018;10(1), a1568. https://doi.org/10.4102/phcfm.v10i1.1568, for more information.

## Strengths and weaknesses

The weakness of this study is the small sample size, especially the low numbers of FPs. Furthermore, the seven district hospitals situated in the Cape Winelands and Overberg districts are not necessarily representative of the whole group of Western Cape district hospitals. Nevertheless, important statistical and clinical information can be inferred regarding the plight of the doctors we studied, whose predicament appears to be similar to medical personnel in other district hospitals.

## Conclusions

This study has shown that there is a high level of burnout among doctors working in rural hospitals in the Cape Winelands and Overberg districts of the Western Cape, similar to doctors working at primary care facilities in the Cape Metropole. The goals of clinician wellness, patient-centred care and better patient outcomes as described in the Health Care 2030 plan of the Western Cape Health are achievable only if the working environments of doctors are improved by reducing workloads, improving working conditions, granting doctors more professional autonomy and by enabling doctors to cope better with the stress related to their occupation. Improvements in working hours and staffing are regrettably long overdue, necessitating a short-term focus on strategies that aim to increase resilience among clinicians by developing coping mechanisms. However, a long-term solution can only be achieved by interventions aimed at improving the work environment. Such interventions should reduce burnout among clinicians, thereby delaying their exit from the public service.
